# Decreased Photosynthetic Efficiency in *Nicotiana tabacum* L. under Transient Heat Stress

**DOI:** 10.3390/plants13030395

**Published:** 2024-01-29

**Authors:** Renan Falcioni, Marcelo Luiz Chicati, Roney Berti de Oliveira, Werner Camargos Antunes, Mirza Hasanuzzaman, José A. M. Demattê, Marcos Rafael Nanni

**Affiliations:** 1 Department of Agronomy, State University of Maringá, Av. Colombo, 5790, Maringá 87020-900, PR, Brazil; mlchicati@uem.br (M.L.C.); rboliveira@uem.br (R.B.d.O.); wcantunes@uem.br (W.C.A.); mrnanni@uem.br (M.R.N.); 2 Department of Biotechnology, Genetic and Cellular Biology, State University of Maringá, Av. Colombo, 5790, Maringá 87020-900, PR, Brazil; 3 Department of Agronomy, Sher-e-Bangla Agricultural University, Dhaka 1207, Bangladesh; mhzsauag@yahoo.com; 4 Department of Soil Science, Luiz de Queiroz College of Agriculture, University of São Paulo, Av. Pádua Dias, 11, Piracicaba 13418-260, SP, Brazil; jamdemat@usp.br

**Keywords:** chlorophyll fluorescence, dark respiration, electron transport chain, gas exchange, nonphotochemical quenching, plant stress, thermal conditions

## Abstract

Heat stress is an abiotic factor that affects the photosynthetic parameters of plants. In this study, we examined the photosynthetic mechanisms underlying the rapid response of tobacco plants to heat stress in a controlled environment. To evaluate transient heat stress conditions, changes in photochemical, carboxylative, and fluorescence efficiencies were measured using an infrared gas analyser (IRGA Licor 6800) coupled with chlorophyll a fluorescence measurements. Our findings indicated that significant disruptions in the photosynthetic machinery occurred at 45 °C for 6 h following transient heat treatment, as explained by 76.2% in the principal component analysis. The photosynthetic mechanism analysis revealed that the dark respiration rate (Rd and Rd^*^_CO2_) increased, indicating a reduced potential for carbon fixation during plant growth and development. When the light compensation point (LCP) increased as the light saturation point (LSP) decreased, this indicated potential damage to the photosystem membrane of the thylakoids. Other photosynthetic parameters, such as *A*_MAX_, *VC*_MAX_, *J*_MAX_, and ΦCO_2_, also decreased, compromising both photochemical and carboxylative efficiencies in the Calvin–Benson cycle. The energy dissipation mechanism, as indicated by the NPQ, qN, and thermal values, suggested that a photoprotective strategy may have been employed. However, the observed transitory damage was a result of disruption of the electron transport rate (ETR) between the PSII and PSI photosystems, which was initially caused by high temperatures. Our study highlights the impact of rapid temperature changes on plant physiology and the potential acclimatisation mechanisms under rapid heat stress. Future research should focus on exploring the adaptive mechanisms involved in distinguishing mutants to improve crop resilience against environmental stressors.

## 1. Introduction

Rising global temperatures, a consequence of climate change, are exerting increasing pressure on agricultural and natural systems worldwide [1]. Plants, which are central to these systems, are highly sensitive to temperature fluctuations, which can lead to significant physiological and metabolic changes such as altered growth patterns and impaired nutrient uptake [2,3]. Despite the general vulnerability of plant structures and functions to high temperatures, the specific mechanisms underlying these responses remain under-researched and require thorough investigation [3,4,5]. Plant heat stress, resulting from temperature instability and daily seasonal fluctuations, disrupts normal physiological processes and reduces productivity and carbon fixation through the Calvin–Benson cycle [4,5,6]. This leads to various morphological, physiological, and structural changes in plant leaves, reflecting the complex challenges that plants face due to abiotic stressors [7,8].

Exposure to elevated temperatures frequently leads to reduced plant growth, molecular changes, and the interruption of essential photosynthetic processes [9]. In higher plants, most tissues undergoing active growth cannot survive prolonged exposure to temperatures above 45 °C or even brief exposure to temperatures of 55 °C or higher [9,10,11,12]. For example, increases in leaf temperature during the day may be more pronounced in plants subjected to desiccation and high irradiance from direct sunlight. Limited air circulation also reduces the rate of evaporative cooling in leaves. Specifically, rapid transient disruptions can occur within chloroplasts and thylakoid membranes as well as during photosynthesis under high-temperature conditions [13]. The trade-off between changes in plant health and organelle adaptive/acclimatization responses to thermal variation remains a focal point of research [9,10,11,12]. Typically, such thermal imbalances reduce the hydric status of cellular growth, impair photosynthetic activity, destabilise proteins through denaturation, and increase the production of reactive oxygen species (ROS), resulting in early oxidative cellular damage and apoptosis over a longer period [9,10,14,15,16,17].

Historical studies have highlighted the long-term effects of thermal stress on plants with a particular focus on the inhibition of photosynthetic (chloroplastidic) processes and alterations in respiratory (mitochondrial) mechanisms [8,9,18,19,20]. However, there is an increasing interest in understanding the immediate responses of plants to short-lived thermal stress events. This rapid physiological acclimation offers insights into plant defences, photosynthetic mechanisms, and resilience to environmental stresses [7,9,17,21].

Recent studies have focused on the effects of heat stress on the photosynthetic machinery, revealing significant effects [8,9,18,19,20,22]. These findings underscore the roles of heat shock proteins (HSPs) and heat shock factors (HSFs) in the electron transport chain under heat stress [1,23,24,25]. For instance, HSPs assist in maintaining the protein structure and preventing denaturation under extreme temperatures. For example, heat stress compromises chlorophyll synthesis, which subsequently affects photosynthesis and the electron transport chain. This phenomenon has been observed in various species such as Arabidopsis [23], pea [26], soybean [18], maize [27], and tomato [28]. Furthermore, photosystem II (PSII) is particularly susceptible to heat, which affects the electron transport rate and ATP synthase activity [15,29,30]. When HSPs and HSFs fail to promote the stability of electron transport chain proteins, they lead to photosynthetic alterations, as identified by fluorescence curves, such as nonphotochemical quenching (NPQ), photochemical quenching (qP), and nonphotochemical quenching due to state transitions (qN) [4,30,31].

Although several studies have shed light on photosynthesis, respiration, and defence mechanisms involved in acclimatisation and adapting conditions to thermal stress, there is still a gap in understanding the initial and rapid transient physiological responses [8,9,18,19,20,22]. Both inter- and intracellular responses in leaves play pivotal roles in of heat stress [22,32]. For example, intercellular signalling is crucial for detecting and modulating changes in various photosynthetic properties induced by membrane receptors to mitigate thermal stress. Conversely, intracellular signalling orchestrates chloroplast interactions, facilitating plant adaptation to temperature fluctuations.

In this study, we aimed to explain the quick photosynthetic alteration induced by heat stress in tobacco plants by delving deeper than PSII activity. By performing real-time photochemical, carboxylative, and fluorescence analyses, we sought to understand the mechanisms of plant acclimatisation strategies, focusing on rapid transient responses to heat stress.

## 2. Results

### 2.1. Photochemical, Carboxylative and Fluorescence Changes in Leaves

**Figure 1:**
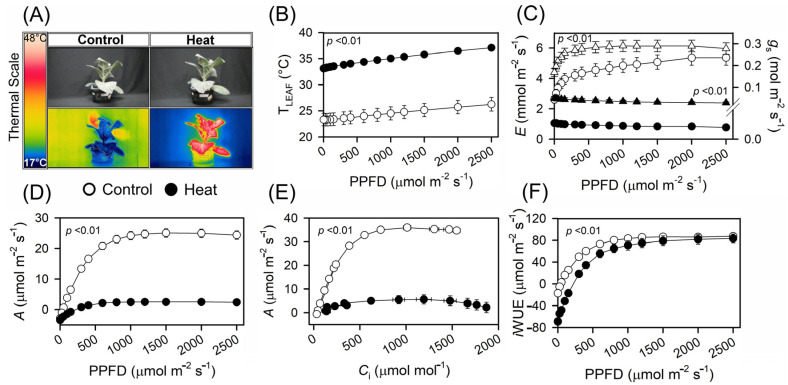
Response curves for light, CO_2_, and fluorescence from tobacco plants under control and heat stress conditions. (**A**) Thermal images of plants under normal and heat stress conditions. The colour gradient, ranging from blue to red, indicates an increase in the temperature. (**B**) The temperature of the leaves was increased from 0 to 2500 μmol m^−2^ s^−1^ PPFD. (**C**) Transpiration rate (*E*) and stomatal conductance (*g*_s_). The white circle represents transpiration (*E*) in the control plants, the white triangle represents stomatal conductance (*g*_s_) in the control plants, the black circle represents transpiration (*E*) under heat stress plants, and the black triangle represents stomatal conductance (*g*_s_) under heat stress plants. (**D**) Net photosynthetic light (*A*-PPFD) response. (**E**) Net photosynthetic CO_2_ (*A*-*C*_i_) response. (**F**) Intrinsic water use efficiency (*i*WUE) response curves. Statistically significant differences according to the *t*-test (*p* < 0.01). Mean ± SE. (*n* = 10).

After exposure to thermal stress at 45 °C for 6 h (Figure 1A), the tobacco plants exhibited physiological and photochemical changes compared to those of the control plants (Figure 1B). In terms of photochemical responses, there was a significant change in the dark respiration rate (Rd; μmol CO_2_ m^−2^ s^−1^). It increased by approximately 37% with values increasing from 2.7 in the control plants to 3.7 (*p* < 0.01) in their heat-treated counterparts (Figure 1C and Table 1). The light compensation point (LCP; μmol photons m^−2^ s^−1^) markedly increased by 586% from 40.3 under control conditions to 276.5 after thermal treatment. In contrast, the light saturation point (LSP; μmol photons m^−2^ s^−1^) decreased by approximately 59%, decreasing from 925.2 in the control plants to 382.7 under heat stress conditions (Figure 1D,E and Table 1).

Furthermore, the net photosynthesis rate (*Pn*_MAX_; μmol CO_2_ m^−2^ s^−1^) showed a significant difference (*p* < 0.01) of 94%: from 23.6 under control conditions to a minor value of 1.4 under thermal stress (Table 1). Similarly, the maximum photosynthetic potential (*A*_MAX_; μmol CO_2_ m^−2^ s^−1^) decreased by approximately 77% with values of approximately 29.0 in the control group and 6.6% in the control and heat-treated plants (Figure 1D,F and Table 1). The maximum quantum yield of photosynthesis (α; (μmol CO_2_ m^−2^ s^−1^)/(μmol photons m^−2^ s^−1^)) also decreased by approximately 57%, changing from a value of 0.07 in the control group to 0.03 under elevated temperature conditions (Figure 1D,E and Table 1). In line with these results, the intrinsic water use efficiency (*i*WUE; (μmol CO_2_ m^−2^ s^−1^)/(μmol photons m^−2^ s^−1^)) decreased by approximately 31%: from 89.4 under control conditions to 61.4 under thermal stress (Figure 1F and Table 1).

### 2.2. Changes in Carboxylative Efficiency

**Figure 2:**
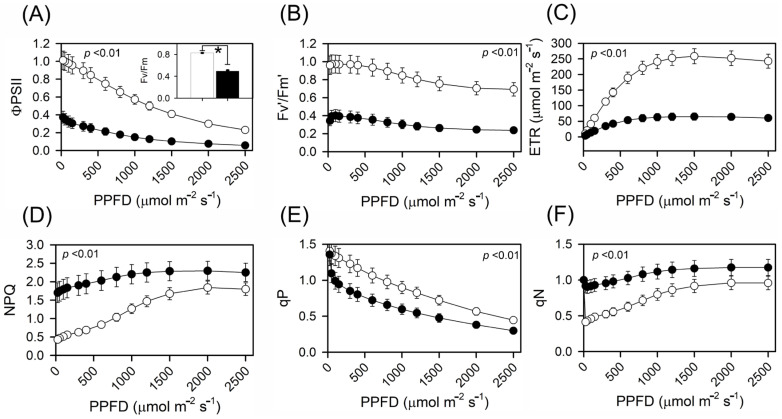
Response curves for chlorophyll a fluorescence in tobacco plants under control and heat stress conditions. (**A**) Operational efficiency of photosystem II (ΦPSII). The inset in the bar graph indicates the maximum quantum yield of PSII (Fv/Fm) in the dark-adapted leaves. (**B**) Effective quantum yield of PSII (Fv/Fm). (**C**) Electron transport rate (ETR). (**D**) Nonphotochemical quenching (NPQ). (**E**) Photochemical dissipation quenching (qP). (**F**) Nonphotochemical dissipation quenching (qN). The white circle represents the control plants and black circle represents under heat stress plants. Asterisks over the bars indicate statistically significant differences according to *t*-test (*p* < 0.01). Mean ± SE. (*n* = 10).

Regarding carboxylative responses, daily respiration (Rd^*^_CO2_; μmol CO_2_ m^−2^ s^−1^) increased by approximately 9167%: from 0.015 in control plants to 1.390 in heat-stressed plants (Figure 2A and Table 1). The maximum carboxylation rate of Rubisco (*VC*_MAX_; μmol CO_2_ m^−2^ s^−1^) significantly decreased by ≈93%: from 156.6 in the controls to 10.7 under heat thermal conditions (Table 1). Simultaneously, the maximum rate of triose phosphate use (TPU; μmol CO_2_ m^−2^ s^−1^) was slightly adjusted downwards by 14%, transitioning from 36.7 in the controls to 31.5 under heat stress (Figure 1D,E and Table 1). Furthermore, the maximum rate of electron transport for a given light intensity (*J*_MAX_; μmol CO_2_ m^−2^ s^−1^) decreased by approximately 78%, changing from 192.8 in the control to 42.1 in the heat-stressed plants (Table 1). Notably, both stomatal conductance (*g*_s_; μmol CO_2_ m^−2^ s^−1^) and mesophyll conductance in response to CO_2_ diffusively (*g*_m_; μmol CO_2_ m^−2^ s^−1^) showed reductions of 85% and 48%, respectively, with values ranging from 0.26 and 0.67 in the control plants to 0.04 and 0.35 in the plants under heat stress (Figure 1C,E and Table 1). In contrast, the chloroplast conductance to CO_2_ transfer (*Cc*; μmol CO_2_ m^−2^ s^−1^) increased by approximately 77%: from 430 μmol CO_2_ m^−2^ s^−1^ in the control conditions to 762 μmol CO_2_ m^−2^ s^−1^ in the thermally treated plants (Figure 1A and Table 1).

### 2.3. Changes in Fluorescence Parameters in Plants

Finally, when the fluorescence response was measured at an intensity of 500 μmol m^−2^ s^−1^, the effective quantum yield of PSII (Fv′/Fm′) decreased by 62%, decreasing from 0.88 in the control group to 0.33 in the heat-treated samples (Figure 2A,B and Table 1). The electron transport rate (ETR; μmol photons m^−2^ s^−1^) also decreased significantly, starting at 157.5 in the control group and decreasing to 42.5 under heat conditions (Figure 2C and Table 1).

**Figure 3:**
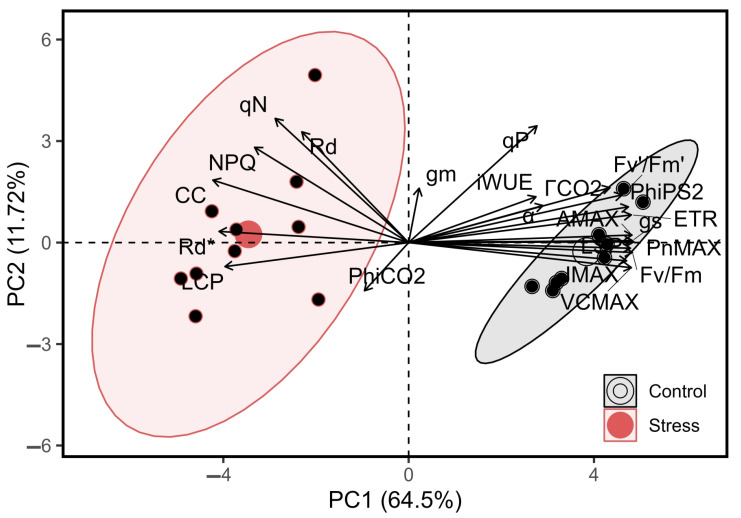
Multivariate analysis of control and stress tobacco plants. The 2D PCA biplot of principal component analysis (PCA) displayed two principal components (PC1 and PC2) and the contribution of the most responsive vectors.

The NPQ (Figure 2D) and (qN) (Figure 2E) values were 1.02 and 0.72, respectively, and the control plants exhibited an increase in the PPFD. However, under heat stress, these values shifted to 2.03 and 1.06 (Figure 2 and Figure 3, Table 1). Similarly, the photochemical dissipation quenching (qP) rate (Figure 2E) decreased from 1.02 in the control plants to 0.75 in the heat-treated plants (Figure 2 and Table 1). Moreover, the operational efficiency of photosystem II (ΦPSII) and the operational efficiency of photosystem II under CO_2_ (ΦCO_2_) decreased substantially from 0.70 in the control group to only 0.21 when subjected to thermal conditions (Figure 2A(inset) and Figure 3 and Table 1).

### 2.4. Principal Component Analysis

**Figure 4:**
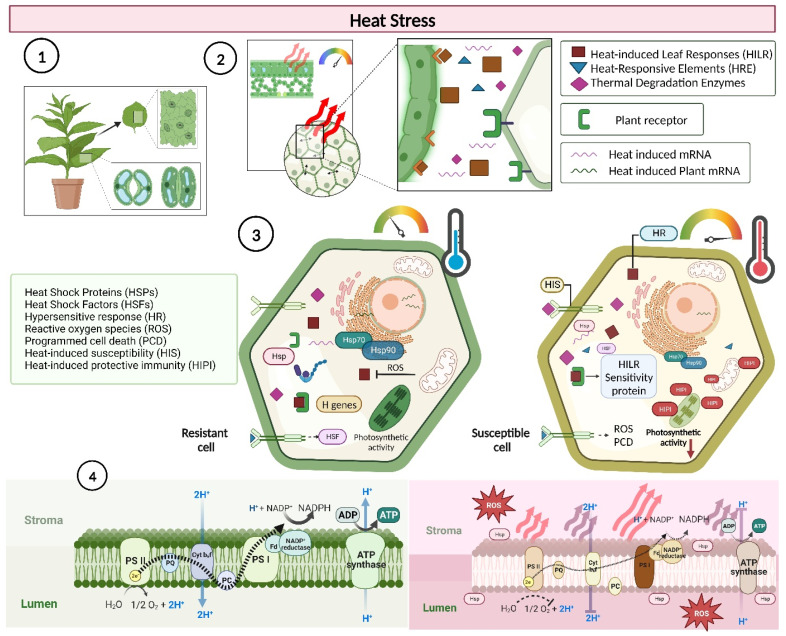
Putative mechanisms underlying the effects of thermal stress on photosynthetic and intra- and intercellular responses in leaves. Heat stress (indicated in red) triggers a series of cellular responses, including photochemical, carboxylative, and fluorescence changes. This leads to alterations in transpiration and stomatal opening/closing as well as intracellular signalling cascades that adjust photosynthetic rates. Intracellular communication within the leaf arranges chloroplast crosstalk, adapting the plant to thermal fluctuations and modulating cellular and photosynthetic activity in chloroplasts. If transient heat modulation does not occur, the electron transport chain may become compromised, leading to reduction and disintegration at the electron transport chain level in the thylakoids. Heat stress can also induce the expression of heat shock proteins (HSPs), heat shock factors (HSFs), hypersensitive responses (HR), reactive oxygen species (ROS), programmed cell death (PCD), heat-induced susceptibility (HIS), and heat-induced protective immunity (HIPI). ROS were generated under thermal stress. (1) Tobacco plants under transient heat stress trigger intercellular and intracellular thermal responses in their leaves. (2) Intercellular signalling is required for the recognition of photochemical, carboxylative, and fluorescence alterations due to heat stress, the initiation of transpiration, stomatal opening/closing, and intracellular signalling cascades to adjust photosynthetic rates. (3) Intracellular signalling within the leaf orchestrates chloroplast crosstalk, driving plant adaptation to thermal fluctuations and consequently modulating cell and photosynthetic activity in chloroplasts. (4) The electron transport chain is compromised if early modulation does not occur, leading to reduction and disintegration at the level of the electron transport chain in thylakoids. The sizes of the arrows indicate the efficiency of the electron transport chain in thylakoid membranes. The figure legends were created using https://www.biorender.com (accessed on 26 December 2023).

A principal component analysis (PCA) optimises the variance of a linear combination of variables by identifying a principal component (PCs—PC1 and PC2) along which observations are maximally separated based on their scores, thus providing a single scale with unequal weights to delineate treatments (Figure 1, Figure 2, Figure 3 and Figure 4 and Table 1). PCA was also conducted to investigate the relationships between the variables most responsive to and correlated with both the control and stress treatment in relation to photochemical and carboxylative efficiency in tobacco plants. The first two principal components accounted for 76.22% of the total variance with PC1 and PC2 explaining 64.50% and 11.72%, respectively (Figure 3).

The control plants treatment was more strongly associated with photochemical, carboxylative, and fluorescence parameters as well as with CO_2_ efficiency. The cluster formed for control was more concentrated; however, among the top 24 variables, 15 were strongly correlated with this control (Figure 3; light-grey clustering). On the other hand, the stressed plants exhibited greater associations with parameters derived from the respiration rate and fluorescence dissipation parameters, such as nonphotochemical quenching. Seven variables were most strongly correlated with the stressed plants and exhibited more compact clustering (Figure 3; light-red clustering). For example, the PCA findings suggest that there are differences in the core physiological performance between these two treatments (Figure 3 and Figure 4).

## 3. Discussion

### 3.1. Rapid Heat Stresses Modulate Photochemical, Carboxylative, and Fluorescence Parameters in Plants

The physiological, biochemical, and molecular changes observed in tobacco plants subjected to high thermal stress significantly affect their photosynthetic processes. Aligned with the literature, these alterations demonstrate how elevated temperatures disrupt the photosynthetic machinery, affecting photochemical, carboxylative, and fluorescence parameters [1,3,8]. The rapidity of physiological adjustments in response to initial thermal stress highlights the importance of immediate plant responses to temperature change. While previous studies have often focused on long-term temperature effects [1,3,8], our results reveal that immediate transient responses to thermal stress are crucial (Figure 1, Figure 2, Figure 3 and Figure 4 and Table 1), potentially even more so than prolonged effects. This indicates the critical need to understand the mechanistic basis of these rapid adaptations, as they play a vital role in plant survival and functionality under abrupt thermal changes.

A significant increase in the dark respiration rate (Rd) was observed under thermal stress [19,33]. This increase in Rd may divert carbon away from growth, potentially reducing the biomass accumulation [18,34]. Such an increase can also be linked to diminished enzymatic activities in the Calvin–Benson cycle at elevated temperatures (Figure 1C–E). Moreover, the increased oxygenating ability of RuBisCO may further reduce fixed carbon gains (Figure 1C, Figure 2, Figure 3 and Figure 4) [8,18]. This process can be influenced by distinct intracellular signalling pathways. However, the enhanced expression of transcription factors associated with heat shock proteins (HSPs) potentially supports the protective effect of RuBisCO [3,10,17,22]. Consequently, these proteins may play a protective role in the chloroplasts and mitochondria over short durations (Figure 3). This transcriptional activation triggers a signalling cascade that prepares the photosynthetic apparatus for adaptive changes, particularly within the electron transport chain (Figure 1, Figure 2, Figure 3 and Figure 4).

The temperature at which the amount of CO_2_ fixed through photosynthesis equals the amount of CO_2_ released by respiration over a specific time period is termed the temperature compensation point. At temperatures exceeding this point, photosynthesis is unable to replenish the carbon consumed as a substrate for respiration. Consequently, carbohydrate reserves are depleted. This imbalance between photosynthesis and respiration is a key factor contributing to the detrimental effects of high temperatures [8,9,18,19,20,22,24].

For instance, a heightened light compensation point (LCP) suggests that plants under thermal stress conditions (heat or cold) require more light to maintain a consistent photosynthetic rate [1,8,9,17]. This adjustment can mitigate the amplified respiratory and photorespiratory effects observed during thermal stress [20,34,35]. This reduces their efficiency, particularly under suboptimal light conditions [36]. This shift may stem from disturbances in the photosynthetic electron transport chain, as has been reported in other studies [8,9,18,19,20,22,24]. Notably, when the LCP increased, the light saturation point (LSP) decreased under thermal stress, respectively (Figure 1D and Table 1). This suggests that plants reach their maximum photosynthetic potential under reduced light intensities [37,38]. In addition, these findings suggest that the photosystems may have been damaged, leading to a decrease in the overall efficiency of light use, as well as a reduction in the production of reducing power, which is generated through cyclic electron flow between PSI, ferredoxin, and ATP synthase [39,40]. The pronounced increase in LCP, aligned with the decrease in LSP following short-term thermal stress, suggested disruptions in the antenna complex and disorganisation of the thylakoid membranes due to thermal dissipation [17,34]. The goal of this mechanism is to mitigate photoinhibitory damage or divert absorbed energy elsewhere, thereby minimising potential harm to the damaged photosynthetic apparatus [3,41,42].

The severe decreases in *Pn*_MAX_, *A*_MAX_, and α suggested that the overall photosynthetic capacity of the plants was compromised [34]. Previous studies have highlighted the vulnerability of RuBisCO to elevated temperatures, which could lead to a reduction in carboxylation efficiency and, subsequently, a decrease in photosynthesis rates [9,18]. Reductions in α reflect a plant’s diminished efficiency in terms of light utilisation for photosynthesis [5,8,43]. Reduced photosynthetic output can also arise from stress-induced stomatal closure, diminished canopy area, and the regulation of assimilate partitioning. Additionally, the decrease in *i*WUE suggested that for every unit of water used, less carbon is fixed in heat-stressed plants [44,45,46]. For example, in terms of carboxylative responses, an increase in day respiration (Rd^*^_CO2_) may be indicative of significant respiratory carbon loss, which is consistent with previous observations in other plant species under transient heat stress [44,45].

### 3.2. Rapid Heat Stresses Decrease the CO_2_ Diffusion and Carboxylation Efficiency of RuBisCO and the Calvin–Benson Cycle

While an increase in temperature augments the catalytic process of RuBisCO, its intrinsic low CO_2_ affinity coupled with its bifunctional role as both carboxylase and oxygenase may limit the potential enhancement of net photosynthesis at increased temperatures [46,47,48]. Notably, when benchmarked against the anticipated outcomes determined by RuBisCO kinetic constraints, empirical data from crops such as cotton suggest the direct curtailment of photosynthesis at temperatures exceeding 35 °C or 49–52 °C for lethality [35,42,49,50]. This highlights, especially in the context of moderate thermal stress ranging between 30 and 42 °C, is largely attributed to the decreasing rates of RuBP regeneration, stemming from perturbations in electron transport dynamics and the specific excitation of photosystem II to the oxygen-evolving complex [1,3,4,9,12,44,45]. Furthermore, sedoheptulose-1,7-bisphosphatase (SBPase) is considered a limiting factor in RuBP regeneration (TPU) within the Calvin–Benson cycle (Figure 1E,F), especially when ATP or NADPH are abundant and under stress conditions, such as elevated CO_2_ concentrations and increased temperatures [13,51]. Elevating SBPase expression in transgenic tobacco plants significantly improved both photosynthetic efficiency and biomass production (Figure 4). Given the impact of heat stress on photosynthesis, it is plausible that this enzyme could positively regulate the Calvin–Benson cycle under such conditions [6,13,20,52]. These findings emphasise the critical role of SBPase in carbon fixation efficiency and highlight the potential advantages of enhancing SBPase activity in agricultural species [13,51].

However, contrasting analyses of chlorophyll fluorescence and ambient CO_2_ metabolite levels offer a paradigm shift, negating the role of electron transport as the primary bottleneck at such inhibitory temperatures [9,13,41]. However, contemporary findings indicate that the impediment to net photosynthesis pivots around the waning activation state of RuBisCO, which is a trend apparent in both C_3_ and C_4_ plants [17]. This reduction in RuBisCO activity is central to the photosynthetic response of plants to temperature [9,18]. However, the biochemical analysis of RuBisCO revealed a sequence of inactivation events. Initially, the accelerated deactivation was caused by the swift formation of dead-end products. Subsequently, reactivation was slowed by activase. This enzyme exhibits redundancy without any functional advantages, thereby consuming large amounts of energy. Consequently, the available TPU and RuBP decreased because of the decreased ETR in the thylakoid membranes and decreased NADPH and ATP production (Figure 2C and Figure 3), particularly under rapid transient heat conditions [9,13]. Therefore, with increasing temperature, the efficacy of activases in sustaining RuBisCO catalytic activity diminishes [9,53,54]. In tandem with these observations, the discernible decreases in *VC*_MAX_ and *J*_MAX_ highlight the thermal sensitivity of RuBisCO and the constraints on the electron transport capacity, respectively. Of particular concern are the reductions in *g*_s_ and *g*_m_ (Figure 1C and Figure 2 and Table 1), which are crucial parameters for CO_2_ diffusion from the ambient atmosphere to the catalytic sites of carboxylation. Such limitations could foreshadow a compromised CO_2_ reservoir, thereby further modulating photosynthesis and carbon loss (Figure 1, Figure 2 and Figure 3 and Table 1).

Under high temperatures, membranes may become destabilized due to increased membrane fluidity. As the lipids in the membrane become more fluid at elevated temperatures, their protein components can no longer function effectively. This leads to the inhibition of numerous biochemical processes, including H^+^ pumping ATPase activity, solute transport into and out of cells, energy transduction, and alterations in chloroplast and thylakoid membrane levels, impacting the entire electron transport chain [9,53,54].

### 3.3. Putative Model for Heat Shock Proteins and Their Role in Chloroplast Stabilisation and Photosynthetic Rates

Recent investigations into tobacco have increased our understanding of its photosynthetic, respiratory, and acclimation processes [4,9,54,55,56,57]. However, the specifics of swift acclimatisation to thermal stress warrant further investigation (Figure 1, Figure 2 and Figure 3 and Table 1). Notably, photosystem II (PSII) demonstrates vulnerability to elevated temperatures in plants, as evidenced by the reduced photochemical efficiency of plants during early thermal changes [38,41,58,59]. Such temperature-induced disturbances lead to modifications in the chloroplast stroma and thylakoids, subsequently affecting chlorophyll levels, electron transfer, and the synergy between PSII and PSI [41,59]. These shifts have implications for carbon dioxide assimilation, potentially skewing the equilibrium between the plant’s source and sink activities [8,18,44].

Certain proteins and transcription factors play pivotal roles in heat stress. Within chloroplasts, small heat shock proteins (HSPs) and heat shock factors (HSFs) are essential for maintaining the stability of proteins and enzymes [3,10,17,60]. At the cellular level, increased temperature amplifies hypersensitive responses (HRs), sometimes leading to programmed cell death (PCD) triggered by an increase in reactive oxygen species (ROS) (Figure 4) [1,61]. Remarkably, chloroplast functions, especially those associated with PSII, may correlate with these cellular responses. Plants may also exhibit heat-induced sensitivities (HIS) or, in certain scenarios, may evolve a heat-induced protective mechanism (HIPI) when initially exposed to thermal extremes (Figure 4). Recent studies have shown that by controlling the nuclear expression of psbA, HSP35, HSP25, and HSP22 under a heat-responsive promoter, plants such as Arabidopsis, tobacco, and rice exhibit increased D1 and PSII levels, PSII repair, thermal resilience in thylakoid membranes, putative mechanisms for CO_2_ assimilation, and the use of CO_2_ (ΦCO_2_) in chloroplasts (*Cc*; Table 1), which ultimately leads to increased biomass and grain yield in rice and other agronomic crops [23,34,62,63,64,65]. In contrast to the earlier belief that these proteins protect (without repairing) photosynthetic electron transport only during stress, specifically by mutants (OEC, PsbP and PsbQ, PsrP, PSII-Mn) and molecules such as kaolin particles which interact with the oxygen-evolving complex proteins of photosystem II (PSII) within the thylakoid lumen, it is now understood that these proteins may aid in repair mechanisms, especially during transient stress (Figure 3 and Figure 4) [46,60,66].

### 3.4. Modulation of Quenching Mechanisms in Response to Heat Stress in Tobacco Leaves

Based on these changes, the fluorescence parameters highlighted the underlying photoprotective mechanisms and reinforced their importance. While the Fv′/Fm′ ratio declined, indicating a decreased photosynthetic efficiency, the increase in NPQ suggested an increase in energy dissipation mechanisms (Figure 2D,F), which is a potential strategy to rapidly prevent photodamage under stress [67,68,69]. Moreover, the pronounced increase in NPQ suggested that a robust photoprotective mechanism was activated early in the plant, which was an attempt by the plant to dissipate excess energy and prevent harm to PSII [23,41].

Nonphotochemical quenching (NPQ) was also associated with light absorption not coupled with electron loss at P680, during which a notably high rate of heat loss from PSII was observed (Figure 2D, Figure 3 and Figure 4) [23,41]. Furthermore, increased NPQ prevents damage to photosystem II by dissipating excess energy as heat. This NPQ phenomenon, as discussed by [67,68,69], is a protective mechanism in plants that dissipates excess light energy as heat, thereby safeguarding PSII from potential damage due to energy overload [23,41]. NPQ is correlated with an enhanced protection of PSII under thermal stress conditions, suggesting that NPQ plays a significant role in maintaining photosynthetic integrity under adverse conditions; hence, increased NPQ is associated with increased heat stress. The rapid activation of photoprotective mechanisms may represent an adaptive strategy for tobacco plants in fluctuating environments, leading to unexpected thermal stress (Figure 1A, Figure 2 and Figure 3).

Alterations in qP and qN substantiate a reconfiguration of the energy distribution within the photosystems [17,18,23,45,70]. A marked decrease in ΦPSII accentuates the adverse effect of thermal stress on PSII photochemical proficiency [38,62,71]. Such a downturn might arise from damage to the reaction centres or the potential disassociation of the antenna complexes from these centres [44,71]. In contrast to the control and heat shock conditions, sustained warming resulted in a reduced photosynthetic rate, which ultimately affected the electron transport rate (Figure 1D and Figure 3). This prolonged thermal stress notably influences qP and qN, diminishing qP and augmenting qN [4,23]. Consequently, our data revealed a significant difference in qP and qN between the control and heat shock conditions in the electron transport chain with a change observed under transient heat in tobacco plants (Figure 2A–F and Figure 3 and Table 1).

In conclusion, our study provides a comprehensive perspective of the diverse implications of thermal stress on tobacco photosynthetic mechanisms through photochemical, carboxylative, and fluorescence changes, highlighting the intricate equilibrium that these plants need to maintain under changing climatic conditions. Ultimately, understanding these rapid responses could pave the way for bolstering thermal tolerance in crucial agricultural crops (Figure 4).

## 4. Material and Methods

### 4.1. Experimental Design and Plant Growth Conditions

**Figure 5:**
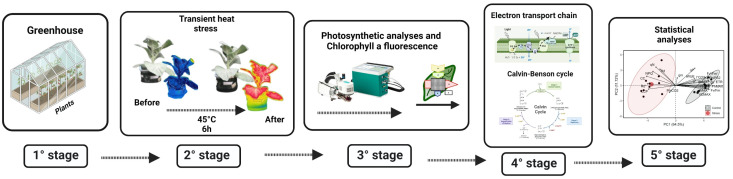
Flowchart of the methodology used for assessing photochemical, carboxylative, and chlorophyll a fluorescence analyses in *Nicotiana tabacum* L. under transient heat stress. 1° Stage: Plants were grown in a greenhouse. 2° Stage: The plants were subjected to transient heat stress at 45 °C overnight for 6 h and evaluated using thermal imaging. 3° Stage: Photosynthetic and chlorophyll a fluorescence analyses were conducted. 4° Stage: The directional flow of electrons in the electron transport chain and the flow of carbon in the Calvin–Benson cycle were evaluated. 5° Stage: Statistical analyses were performed, including principal component analysis. * shows the flow of electrons.

Tobacco plants (*Nicotiana tabacum* L. cv. Samsun-SS) were grown in a greenhouse and a growth chamber under white LED light. Plants were grown in 1 L pots filled with MecPlant^®^ (MecPrec Ind., Telêmaco Borba, PR, Brazil), a commercial substrate, supplemented with NPK (10-10-10; 0.5 g pot^−1^) in a greenhouse under natural lighting conditions. The controlled chamber environment was maintained at a temperature of 22–26 °C and 60–70% humidity following a 16 h light and 8 h dark cycle, respectively. To ensure consistent hydration, the plants were watered twice daily, once at 8 a.m. and again at 6 p.m. After 20 days of growth, one set of plants was kept as a control, whereas another set (comprising 10 samples) underwent thermal stress at 45 °C for 6 h in the absence of light. The temperature and duration were selected based on previous experiments, which determined the lethal threshold for tobacco plants to be between 49 and 51 °C for 10 min. A combined total of 10 plants were analysed before and after heat stress. After the thermal stress treatment, the plants were subjected to photosynthetic analyses, including light (*A*-PPFD), CO_2_ (*A*-*C*_i_), and fluorescence (ChlF) curve analyses, as described in the following sections. Near-infrared (NIR) thermal images of tobacco plants were immediately recorded using a Flir One Pro radiometric thermal camera by FLIR Systems (Danderyd, Sweden) (Figure 5).

### 4.2. Gas Exchange and Fluorescence Analyses

#### 4.2.1. Light Curves with a Multiphase Flash^TM^ Fluorometer

Gas exchange measurements were conducted on healthy, young, fully expanded leaves (typically the 5th or 6th leaf counting downwards from the apical meristem) from experimental plants approximately 20 days after transplantation. An infrared gas exchange analyser (IRGA) (LI-6800, Li-Cor Inc., Lincoln, NE, USA) was used in tandem with a Multiphase Flash^TM^ Fluorometer (LI-6800-01). This setup enabled the determination of the net carbon assimilation rate (*A*), intercellular CO_2_ concentration (*C*_i_), transpiration rate (*E*), and stomatal conductance (*g*_s_). The photosynthetic light response curve was generated using the manufacturer’s light source, which provided a range of photosynthetically active radiation (PPFD) levels (2500, 2000, 1500, 1200, 1000, 800, 600, 400, 300, 150, 100, 50, 25, and 0 µmol m^−2^ s^−1^). Measurements were carried out with a red to blue light ratio of 90:10, steady 400 µmol mol^−1^ CO_2_ concentration in the sample chamber, 60% relative humidity, medium flow rate of 700 µmol s^−1^, fan speed of 10,000 rpm, and a temperature of 25 °C. Fluorescence measurements were performed concurrently with the readings.

The quantum yield of photosynthesis (α) ((µmol CO_2_ m^−2^ s^−1^)/(µmol photon m^−2^ s^−1^)), light compensation point (LCP) (µmol photons m^−2^ s^−1^), light saturation point (LSP) (µmol photons m^−2^ s^−1^), maximum net photosynthetic rate (*A*_MAX_) (µmol CO_2_ m^−2^ s^−1^) and dark respiration rate (Rd) (µmol CO_2_ m^−2^ s^−1^) were estimated using linear (*Y* = *ax* + *b*), hyperbolic models $\left[Y=y0+\frac{\left(ax\right)}{\left(b+x\right)}\right]$ or photosynthesis in relation to light and carbon dioxide $\left[Pn_{MAX}=\frac{[\Phi \left(I0\right)\;\times \;I\;\times \;{\mathrm{Pg}}_{\mathrm{MAX}}]}{{{\left[\Phi (I0\right)}^{2}\;\times \;I^{2}+{{\mathrm{Pg}}_{\mathrm{MAX}}}^{2}]}^{0.5}}-Rd\right]$, where *Pn*_MAX_ = net photosynthesis rate (mmol (CO_2_) m^−2^ s^−1^); Φ(I0)= quantum yield at I = 0 (mmol (CO_2_) mmol^−1^ (photons)); I = photosynthetic photon flux density (mmol (photons) m^−2^ s^−1^); Pg_MAX_ = maximum gross photosynthesis rate (mmol (CO_2_) m^−2^ s^−1^); and Rd^*^_CO2_ = dark respiration rate (mmol (CO_2_) m^−2^ s^−1^). Additionally, we calculated the intrinsic water use efficiency (*i*WUE) using the *A*/*g*_s_ ratio ((μmol m^−2^ s^−1^)/(mol m^−2^ s^−1^)) to account for alterations in leaf structure (mesophyll conductance) and ultrastructure (chloroplast conductance) in the photosynthetic curves.

#### 4.2.2. *A*-C_i_ Curves with a Multiphase Flash^TM^ Fluorometer

Photosynthetic *A*-*C*_i_ response curves were generated, and fluorescence measurements were performed simultaneously. These parameters were used to determine the carboxylation efficiency of the plants. Photosynthetic CO_2_-response (*A*-*C*_i_) curves were measure using CO_2_ chamber reference (CO_2__reference) concentrations (400, 300, 200, 100, 50, 25 400, 600, 800, 1000, 1300, 1600, 1800, 2000 µmol mol^−1^ and fixed light of 1000 µmol m^−2^ s^−1^ PPFD) using a commercial light source (50–70 s, min–max; red:blue ratio (90:10)); 60% sample chamber relative humidity (%RH_sample); and flow 700 µmol s^−1^ with ΔP (0.1) for flow adjustment and VPD constant and automated adjustment by an LICOR 6800; fan speed, 10,000 rpm; and 25 °C heat exchanger temperature. The estimated rates of day respiration (Rd^*^_CO2_; µmol CO_2_ m^−2^ s^−1^), maximum carboxylation rate of Rubisco (*VC*_MAX_; µmol CO_2_ m^−2^ s^−1^), maximum rate of triose phosphate use (TPU; µmol CO_2_ m^−2^ s^−1^), maximum rate of electron transport for the given light intensity (*J*_MAX_; µmol photons m^−2^ s^−1^), stomatal conductance (*g*_s_; µmol CO_2_ s^−1^ mmol^−1^), mesophyll conductance to CO_2_ transfer (*g*_m_; µmol CO_2_ s^−1^ mmol^−1^), and chloroplast conductance to CO_2_ transfer (*Cc*; µmol CO_2_ s^−1^ mmol^−1^) were calculated using the script “PCE_Calculator_Curve_Fitting_Model 2.0” developed for tobacco plants and made available in “Plant Cell & Environment 2016” [72] and RStudio-package software version 2023.06.1 (Posit Software, PBC, Boston, MA, USA) for fit models *A*-*C*_i_ curves. The constants for the equipment parameters were adjusted to a leaf temperature of 25 °C in the sample chamber, an atmospheric pressure (Patm) of 101 kPa, and an O_2_ concentration of 21 kPa [72].

#### 4.2.3. Fluorescence Measurements

Fluorescence measurements were performed using an LI-6800 (Li-Cor Inc.) instrument equipped with a multiphase flash fluorometer (LI-6800-01). The plants were dark acclimated for 12 h (overnight) to measure the “dark acclimated” fluorescence PPFD parameters, initial fluorescence (Fo) and maximum fluorescence (Fm). Variable fluorescence (Fv) was calculated as Fv = Fm − Fo, enabling calculation of the Fv/Fm ratio (maximum quantum yield of PSII in dark-adapted leaves). Additional chlorophyll fluorescence measurements were conducted using “light-acclimated leaves” during the analysis of light response curves. The multiphase flash fluorescence (MPF) protocol was applied with a saturating intensity of 15,000 µmol m^−2^ s^−1^, a dark modulation rate of 5 kHz, and a light modulation rate of 50 kHz for an optimal signal-to-noise ratio. The maximum Chl fluorescence (Fm′) was measured at 250 kHz during the saturating pulse, and fluorescence was detected at wavelengths greater than 700 nm (Li-Cor Inc.). The effective quantum yield of PSII (Fv′/Fm′), operational efficiency of photosystem II (ΦPSII), operational efficiency of photosystem II under CO_2_ (ΦCO_2_), electron transport rate through photosystem II (ETR) (µmol m^−2^ s^−1^), nonphotochemical quenching (NPQ), photochemical dissipation quenching (qP), and nonphotochemical dissipation quenching (qN) were estimated using LI-COR software version 1 in tandem with gas exchange measurements [73].

### 4.3. Statistical Analyses

The homogeneity of variance across all variables was verified using Bartlett’s test, thus negating the need for data transformation. Quantitative data (10 samples) were analysed using paired Student’s *t* tests and are presented as the mean ± standard error (SE). The threshold for statistical significance was set *p* < 0.01. All curve fitting and statistical and image analyses of the parameters were performed using Statistica^®^ 10.0 (StatSoft Inc., Tulsa, OK, USA), SigmaPlot^®^ 10.0 (Systat Software, Inc., San Jose, CA, USA), and the R statistical package [74].

Multivariate analysis of the dataset related to photochemical, carboxylative, and fluorescence parameters was conducted using principal component analysis (PCA) with a significance threshold of *p* < 0.01 applied to ensure the robustness of the analysis. To avoid underfitting and overfitting, the optimal number of principal components was determined based on the first peak value of the cumulative explained variance, as indicated by Zar (2010). Furthermore, PCA was employed to form clusters between the two treatments and vectors associated with each cluster for each parameter measure. This approach provides a comprehensive understanding of photosynthetic parameters and their relationships in plants under both control and heat-stress conditions.

## 5. Conclusions

Understanding transient heat stress responses is important for directing research toward constant increases in environmental temperature. We observed significant changes in the photosynthetic parameters of the plants, indicating their sensitivity to rapid heat stress. Critical heat shock treatment at 45 °C led to a marked decrease in photosynthetic efficiency and impaired carbon fixation via the Calvin–Benson cycle. These findings elucidate the interplay between environmental stressors and photosynthetic processes in plants. Future research should investigate the adaptive mechanisms of stomata, chloroplasts, anthocyanins or the phenolics, and hormones pathway in mutant plants and on the to increase crop resilience. In conclusion, these initial responses in the context of rising heat levels underscore the challenges posed by rapid heat stress in warming climates.

## Figures and Tables

**Table 1:** Estimated photosynthetic and fluorescence parameters in response to light and CO_2_ curves of tobacco plants. The parameters include dark respiration rate (Rd; μmol CO_2_ m^−2^ s^−1^), light compensation point (LCP; μmol photons m^−2^ s^−1^), light saturating point (LSP; μmol photons m^−2^ s^−1^), net photosynthesis rate (*Pn*_MAX_; μmol CO_2_ m^−2^ s^−1^), maximum photosynthetic potential (*A*_MAX_; μmol CO_2_ m^−2^ s^−1^), and maximum quantum yield of photosynthesis [α; (μmol CO_2_ m^−2^ s^−1^)/(μmol photons m^−2^ s^−1^)], intrinsic water use efficiency (*i*WUE; (μmol CO_2_ m^−2^ s^−1^)/(μmol photons m^−2^ s^−1^)), day respiration (Rd^*^_CO2_; μmol CO_2_ m^−2^ s^−1^), maximum carboxylation rate of Rubisco (*VC*_MAX_; μmol CO_2_ m^−2^ s^−1^), maximum rate of triose phosphate use (TPU; μmol CO_2_ m^−2^ s^−1^), maximum rate of electron transport for the given light intensity (*J*_MAX_; μmol CO_2_ m^−2^ s^−1^), stomatal conductance (*g*_s_; μmol CO_2_ m^−2^ s^−1^), mesophyll conductance to CO_2_ transfer (*g*_m_; μmol CO_2_ m^−2^ s^−1^), chloroplast conductance to CO_2_ transfer (*Cc*; μmol CO_2_ m^−2^ s^−1^), effective quantum yield of PSII (Fv′/Fm′), electron transport rate (ETR; μmol photons m^−2^ s^−1^), nonphotochemical quenching (NPQ), photochemical dissipation quenching (qP), nonphotochemical dissipation quenching (qN), operational efficiency of photosystem II (ΦPSII) and operational efficiency of photosystem II under CO_2_ (ΦCO_2_). The estimated fluorescence parameters were obtained at 500 μmol m^−2^ s^−1^. The Fv/Fm values for the maximum quantum yield of PSII in dark-adapted leaves are reported in Figure 2A (inset) to be 0.82 and 0.49. The bold of number indicates statistical significance by the *t*-test (*p* < 0.01). Mean ± SE. (*n* = 10).

Parameters		Control	Heat-Stress
**Photochemical**	**Rd**	2.7 ± 0.23	**3.7 ± 0.27**
	**LCP**	40.3 ± 3.0	**276.5 ± 55.0**
	**LSP**	**925.2 ± 25.8**	382.7 ± 18.9
	***Pn*** **_MAX_**	**23.6 ± 1.54**	1.4 ± 0.57
	***A*** **_MAX_**	**29.0 ± 1.66**	6.6 ± 0.62
	**α**	**0.07 ± 0.004**	0.03 ± 0.011
	***i*** **WUE**	**89.4 ± 2.40**	61.4 ± 6.94
**Carboxylative**	Rd^*^_CO2_	0.015 ± 0.002	**1.39 ± 0.187**
	***VC*** **_MAX_**	**156.6 ± 1.75**	10.7 ± 0.54
	**TPU**	**36.7 ± 0.51**	31.5 ± 0.74
	***J*** **_MAX_**	**192.8 ± 8.90**	42.1 ± 3.40
	***g*** **_s_**	**0.26 ± 0.024**	0.04 ± 0.007
	***g*** **_m_**	**0.67 ± 0.017**	0.35 ± 0.011
	***Cc***	430 ± 25.3	**762 ± 34.5**
**Fluorescence**	**Fv′/Fm′**	**0.88 ± 0.09**	0.33 ± 0.05
	**ETR**	**157.5 ± 14.99**	42.5 ± 7.91
	**NPQ**	1.02 ± 0.10	**2.03 ± 0.26**
	**qP**	**1.03 ± 0.09**	0.75 ± 0.09
	**qN**	0.72 ± 0.06	**1.06 ± 0.10**
	**Φ** **PSII**	**0.70 ± 0.07**	0.21 ± 0.04
	**Φ** **CO_2_**	**0.06 ± 0.01**	0.04 ± 0.01

## Data Availability

Data are contained within the article.
